# A symmetric multivariate leakage correction for MEG connectomes

**DOI:** 10.1016/j.neuroimage.2015.03.071

**Published:** 2015-08-15

**Authors:** G.L. Colclough, M.J. Brookes, S.M. Smith, M.W. Woolrich

**Affiliations:** a Oxford Centre for Human Brain Activity (OHBA), University of Oxford, Oxford, UK; b University of Oxford, Dept. Engineering Sciences, Parks Rd., Oxford, UK; c Centre for the Functional Magnetic Resonance Imaging of the Brain (FMRIB), University of Oxford, Oxford, UK; d Sir Peter Mansfield Magnetic Resonance Centre, School of Physics and Astronomy, University of Nottingham, University Park, Nottingham, UK

## Abstract

Ambiguities in the source reconstruction of magnetoencephalographic (MEG) measurements can cause spurious correlations between estimated source time-courses. In this paper, we propose a symmetric orthogonalisation method to correct for these artificial correlations between a set of multiple regions of interest (ROIs). This process enables the straightforward application of network modelling methods, including partial correlation or multivariate autoregressive modelling, to infer connectomes, or functional networks, from the corrected ROIs. Here, we apply the correction to simulated MEG recordings of simple networks and to a resting-state dataset collected from eight subjects, before computing the partial correlations between power envelopes of the corrected ROItime-courses. We show accurate reconstruction of our simulated networks, and in the analysis of real MEGresting-state connectivity, we find dense bilateral connections within the motor and visual networks, together with longer-range direct fronto-parietal connections.

## Highlights

• A method for removing source leakage from multivariate network analyses in MEG.
• Network inference performed using regularised partial correlations between ROIs.
• Artificial correlations are removed using a symmetric orthogonalisation step.
• Simulations show accurate false-positive rates for network edge detection.
• Resting-state networks show increased bilateral connectivity after correction.

## Introduction

The discovery of robust networks of correlated activity in the human brain, visible in functional imaging data, has triggered much recent work into the functional connectivity of both healthy and dysfunctional brains. Analysis techniques have focussed not just on network discovery, and changes in network activation, but also on mapping the presence, strength and potentially the direction of the connections between brain regions. Within the functional magnetic resonance imaging (fMRI) literature, parcellation-based network modelling has become quite common. First a parcellation, or set of regions of interest (ROIs), is defined using a standard anatomical or histological brain atlas or a data-driven technique such as independent components analysis (ICA, Beckmann etal., 2005). These ROIs are then treated as nodes in a network, and the relationships between them can be analysed leading to graphs of the functional connections between brain regions (connectomes), and comparisons of connection strengths between populations of interest Smith (2012), Woolrich and Stephan (2013).

Magnetoencephalography (MEG) has provided independent corroboration of the existence of functional networks in the human brain Brookes etal. (2011, 2012b), Luckhoo etal. (2012), Hipp etal. (2012), Marzetti etal. (2013), Mantini etal. (2011), de Pasquale etal. (2010), Stam and van Straaten (2012), Wens etal. (2014), but network connectivity analysis in this modality is less mature than in fMRI. Immediate application of the tools used in fMRI to MEG is hampered by the presence of artefactual correlations between the inferred cortical sources. These correlations are a result of the ill-posed inverse problem: the few hundred magnetic field sensors cannot provide sufficient discriminatory information to independently estimate the source activity in thousands of brain voxels. Reconstructions of activity within the brain are coupled over space, with neighbouring regions exhibiting temporally-correlated behaviour. This effect is frequently termed “source leakage” as the reconstructions of true point dipole sources from the measured signals will be spread over several voxels.

MEG connectivity studies which seek to be robust to source leakage artefacts either assess metrics of phase lag between sources, which are insensitive to linear leakage effects Nolte etal. (2004), Stam etal. (2007), Baccala and Sameshima (2001), Kaminski and Blinowska (2014), or attempt to correct for leakage before performing signal amplitude correlations. Authors using the latter technique have so far been limited to pair-wise comparisons of source activities between voxels Brookes etal. (2012a), Hipp etal. (2012). Although this work has recently been extended from the analysis of two voxels to a canonical correlation analysis between a pair of ROIs Brookes etal. (2014), there has so far been no simple technique correcting amplitude correlations between larger collections of brain areas, which limits the scope of whole-brain ROI network analyses.

Source leakage is a linear effect, and it introduces cross-correlations only at zero phase lag. Thus, the correlation artefacts caused by source leakage can be corrected (under certain assumptions) by removing zero-lag correlations between the variables of interest, before performing any connectivity analyses. The existing methodology Brookes etal. (2012a), Hipp etal. (2012) is to orthogonalise reconstructed source time-courses with respect to a seed voxel (which removes their mutual correlation at zero lag), then to analyse frequency-dependent connectivity by correlating the down-sampled power envelopes of the orthogonalised sources within specific frequency bands. As well as being limited to pair-wise comparisons between the seed and the rest of the brain, these correlations are sensitive to which of the two voxels is chosen as the seed. Often a compromise is taken, by averaging the correlations in band-limited power, for each pair of voxels, over the two choices of seed Hipp etal. (2012).

We present a symmetric, multivariate, spatial leakage correction which extends this method to enable corrected multivariate network analyses between multiple ROIs. We remove the effects of signal leakage at zero lag using a symmetric orthogonalisation process over all ROIs which has three valuable properties: firstly, the outcome is not biased by any ordering of the ROI time-courses; secondly, the resulting orthogonal vectors are as close as possible to the original time-courses; lastly, the computation is based on the singular value decomposition, which is a highly optimised routine on most computational platforms. The number of regions which can be corrected is limited only by the dimensionality of the recorded data. Here, we demonstrate the method by modelling the connected ROIs as a Gaussian Markov network, and estimate the edges of the graph (that is, the connections between ROIs) using regularised partial correlations between the band-limited power envelopes of the corrected ROI time-courses. We validate our approach on simulated networks of dipoles within the cortex and on resting-state data from a small sample of healthy subjects.

## Theory

### A symmetric, multivariate correction for signal leakage between ROIs

We characterise the behaviour of each ROI by a single time-course. The particulars of this definition are unimportant here; common methods in fMRI and MEG include taking the mean time-course, or the coefficients of the principal component accounting for the majority of the ROI's variance, some authors in the MEG literature select just the single voxel within the ROI with maximal power (e.g.,Hillebrand etal., 2012).

We correct for signal leakage by removing any correlations with zero temporal lag between all ROIs. We employ a multivariate symmetric orthogonalisation technique Everson (1999), Löwdin (1950), which produces an optimal set of mutually orthogonal time-courses for each ROI: the solution is unique, unaffected by any re-ordering of ROIs and is minimally displaced from the uncorrected ROItime-courses (as measured by the least-squares distance).

**Fig. 1:**
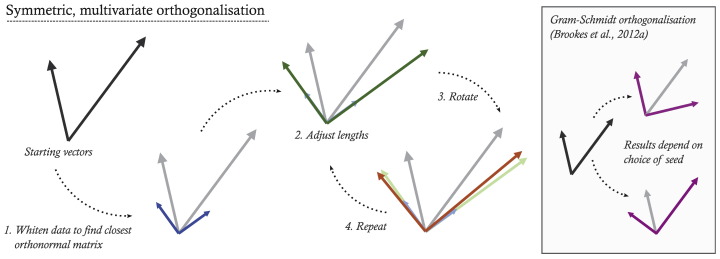
A symmetric, multivariate orthogonalisation process. The correlations between ROIs which are introduced during source reconstruction can be removed by mutually orthogonalising the ROI time-courses, illustrated here for two vectors in two dimensions. We construct an optimal set of corrected time-courses by iterating towards the closest set of orthogonal vectors to the starting time-courses. The process is initialised with the closest orthonormal matrix to the uncorrected vectors, then adjusts in turn the vector magnitudes and orientations to minimise the Euclidean distance between the corrected and uncorrected time-courses. The box, right, shows the result of regressing one vector from the other, with differing outcomes depending on the choice of seed, as used for correcting pairs of voxels in Brookes etal. (2012a).

We construct the corrected time-courses in two stages, illustrated in Fig. 1. Firstly, we find the closest set of orthonormal time-courses, for which there is a simple analytic solution. Secondly, we relax the normality constraint and finesse the solution by iteratively adjusting the lengths and orientations of the corrected vectors until we converge to a solution which is as close as possible to the uncorrected time-courses.

We outline the proof in Everson (1999) here. For a set of *n*ROItime-courses containing *m* time samples ***Z*** = {**z**_1_, **z**_2_, ⋯, **z**_*n*_} ∈ ℝ^*m* × *n*^, *m* ≥ *n*, we seek the matrix of corrected time-courses***P*** which minimises the Frobenius norm

(1) $$\epsilon ={\left\|Z-P\right\|}_{F}^{2}\text{≡}\mathrm{trace}\left[{\left(Z-P\right)}^{T}\left(Z-P\right)\right]\;\text{.}$$

If we constrain ***P*** to contain orthogonal vectors, we can express

(2) P=OD

where ***O***^T^***O*** = ***I***_*n*_ and ***I***_*n*_ is the *n* × *n* identity matrix, ***D*** = diag(***d***) is diagonal, and ***P***^T^***P*** = ***D***^2^. Hence,

(3) $$\epsilon =\mathrm{trace}\left(Z^{T}Z\right)-2\mathrm{trace}\left(Z^{T}OD\right)+\mathrm{trace}\left(D^{2}\right)\;\text{.}$$

If ***D*** is known and ***Z*** is full rank, we minimise *ϵ* by maximising trace(***Z***^T^***OD***), using the singular value decomposition of ***ZD***:

(4) $$\begin{array}{c} ZD=U\Sigma V^{T},\\ \Sigma =\mathrm{diag}\left(\sigma_{1},\sigma_{2}\ldots ,\sigma_{n}\right),\sigma_{i}>0\;\forall i\text{.} \end{array}$$

We bound this expression by noting that all elements of the orthonormal matrix ***T*** = ***U***^T^***OV*** must have a magnitude |*T*_*ij*_| ≤ 1 ∀ (*i*, *j*):

(5) $$\mathrm{trace}\left(Z^{T}OD\right)=\mathrm{trace}\left(T\Sigma \right)\leq \sum_{i=1}^{n}\sigma_{i}\text{.}$$

This is maximal when ***T*** = ***I***_*n*_, yielding

(6) $$O=UV^{T}\text{,}$$

which is the symmetric or Löwdin orthogonalisation Löwdin (1950) of***ZD***.

On the other hand, if ***O*** is known, the optimal ***d*** is given by

(7) $$\frac{\partial \epsilon }{\partial d_{k}}=-2\sum_{i=1}^{m}Z_{ik}O_{ik}+2d_{k}=0$$

which yields

(8) $$d=\mathrm{diag}\left(Z^{T}O\right)\text{.}$$

To find the corrected time-courses ***P***, we follow Everson (1999)'s tandem algorithm by starting with ***D***^(1)^ = ***I***_*n*_, which gives ***P***^(1)^ as the symmetric orthogonalisation of ***Z*** and the unique closest ortho*normal* matrix to the uncorrected ROItime-courses; we then allow the vector magnitudes to vary and reduce the error *ϵ* by iterating (4), (6)and (8) until convergence. Convergence is guaranteed, but not necessarily to a global minimum of *ϵ*. In our data, this procedure tends to converge within twenty iterations.

This orthogonalisation process to find corrected time-courses***P*** by definition removes all correlations between ROIs at zero phase lag. Any remaining correlations between the band-limited power envelopes of these orthogonalised time-courses are thought to represent true biological dependencies (at the expense of true zero-phase-lag connectivity) Brookes etal. (2012a), Hipp etal. (2012), Luckhoo etal. (2012). Our approach is limited by the rank of the data: we cannot correct more ROIs than we have dimensions in ***Z*** as there is no longer a unique solution to (5).

Following Brookes etal. (2012a), we compute power envelopes ofthe corrected ROIs, $\tilde{P}$, as the absolute values of the analytic Hilbert transform of ***P***, which are then low-pass filtered to 0.5 Hz and re-sampled at 1 Hz.

### Network analysis

We estimate the connections between ROIs by modelling the corrected and down-sampled power envelopes with an undirected Gaussian graphical model, $\tilde{P}\sim N_{n}\left(0,\Omega^{-1}\right)$, where **Ω** is the precision or inverse covariance matrix. The network connections between ROIs—the edges of the graph—are estimated from the partial correlation matrix ***ρ***_⊥_, which is the conditional correlation between variables with the effect of all other variables removed

(9) $$\rho_{⊥}=-\mathrm{diag}{\left(\Omega \right)}^{-1/2}\Omega \;\mathrm{diag}{\left(\Omega \right)}^{-1/2}\text{.}$$

Zeros in the partial correlation matrix identify variables which are conditionally independent (unconnected by a network edge), thus partial correlations relate only to direct connections in the network graph. The estimation of partial correlations from a limited dataset is noisy Varoquaux etal. (2010), Varoquaux and Craddock (2013): it is standard practice to regularise the estimate of the precision matrix, $\hat{\Omega }$, and suppress null edges using the graphical lasso Friedman etal. (2008). This is a sparsity promoting procedure which maximises the log-likelihood of the multivariate Gaussian model, subject to a penalty on the *L*_1_ norm of the precision (the sum of the absolute values in **Ω**). The strength of the regularisation penalty is determined by a free parameter λ, which can be chosen by cross-validation:

(10) $$\hat{\Omega }=\max_{\Omega }\left[\log\;\det\Omega -tr\left(\frac{1}{m}{\tilde{P}}^{T}\tilde{P}\Omega \right)-\lambda ||\Omega {\left|\right|}_{1}\right]\text{.}$$

## Methods

We test the efficacy of our approach on both real and simulated resting-state datasets. Data processing and analyses were performed using in-house Matlab scripts, “quadratic programming in C” routines from the University of Newcastle, Australia Ninness etal. (2005), SPM8, FieldTrip Oostenveld etal. (2011) and FSL Jenkinson etal. (2012). We solved the graphical lasso using custom implementations of the algorithms in Mazumder and Hastie (2012a,b).

### Generation of simulated data

**Fig. 2:**
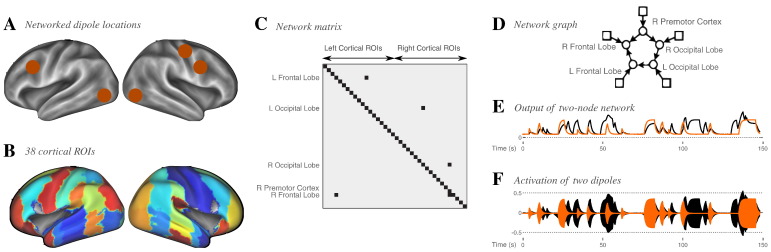
Simulation of a five-dipole network. Five dipoles with differing carrier frequencies in the range 8–26 Hz, modulated in amplitude by a simple five-node network drawn from Smith etal. (2011), were placed into five ROIs of a 38-ROI parcellation of the cortex, and the signals recorded in a set of 306 MEG sensors were simulated with additive measurement noise. Ashows the locations of the five simulated dipoles; B a surface rendering of the 38 ROIs drawn from a laterally-split version of the Harvard–Oxford cortical atlas in FSL; C shows the network matrix used to simulate the activities in the five dipoles, placed within context of all 38 ROIs considered in later analysis; D shows the network as a graph, indicating the directions of the network edges and the Poisson processes stimulating each node independently; E shows example amplitude time-courses from a simpler two-node network, with one node (orange) driving the other (black), and both stimulated by independent Poisson processes; and F displays the modulation of the activations of two dipoles by the amplitudes in E.

Fifteen resting-stateMEG experiments of 600 s were simulated. In each experiment, five equivalent current dipoles were active, positioned in the left and the right frontal gyrus, the left and the right lateral occipital cortex, and the right premotor cortex. Beamforming methods for source reconstruction were used, which have imperfect reconstruction success with sources which are highly linearly-correlated over the whole acquisition Van Veen etal. (1997), Robinson and Vrba (1999), Gross etal. (2001). We therefore simulated each dipole as oscillating with amplitude 1 nAm at a different carrier frequency in the range 8–26 Hz, modulated by a “functional activity” which was generated by a simple 5-node network model drawn from Smith etal. (2011), based on the network model underlying the dynamic causal model for fMRI activity Friston etal. (2003). In brief, amplitudes or activities **a** are generated according to

(11) $$\dot{a}=\mathrm{Aa}+u+e$$

where the network edges are non-zero entries on the off-diagonal elements of **A**, with a strength randomly chosen from $N\left(0.6,0.1\right)$; the diagonal elements of **A** are set to − 1, modelling within-node temporal decay; a set of inputs, **u**, drive each node, modelled by a binary Poisson process^1^ of strength 0.4, with a mean on-time of 2 s and a mean off-time of 7 s; and a Gaussian noise input **e** is drawn from $N\left(0,0.02\right)$. We integrate Eq. (11) using a fourth-order Runge–Kutta method. The network structure is shown in Fig. 2D, together with example activity time-courses and modulated oscillations for a simpler 2-node network in Figs.2E and 2F.

From these dipole sources, MEG data were simulated for the 306 sensors on an Elekta Neuromag MEG instrument (Stockholm, Sweden), at a sampling rate of 150 Hz, using a multiple local sphere volume conductor Huang etal. (1999) and the Sarvas (1987) current dipole forward model. Sensor noise was added by sampling from a zero-mean multivariate Gaussian distribution, using a covariance matrix estimated from 10 min of empty-room recordings. The noise was scaled to produce a unit signal-to-noise power ratio over all the sensors (SNR, calculated as the ratio of the mean-square signal amplitude in all the sensors to the variance of the noise).

A second dataset of fifty experiments was created using the same methodology, but increasing the number of dipoles to 38, chosen to match the number of ROIs used in later analysis. Only five dipoles were networked, using the same structure as above; the rest were also generated from (11) but without any network structure and uncorrelated. Dipoles were randomly located such that each ROI contained one dipole, and the ROIs containing networked dipoles varied between experiments. The simulated SNR in the sensors was 0.4.

### Resting-state data collection and pre-processing

Ten minutes of MEG data were acquired from ten healthy subjects (7males, 3 females of which one was left-handed; 27 ± 0.5 years of age) in a resting-state using a 275-channel CTF whole-head system (DC, Canada) at a sampling rate of 600 Hz with a 150 Hz low-passanti-aliasing filter applied. Subjects were asked to lie in the scanner and view a centrally-presented fixation cross. The study was approved by the University of Nottingham Medical School Research Ethics Committee (approval code F/12/2006). All volunteers received a study information sheet, completed a safety questionnaire, and provided written informed consent, including consent to publish anonymised results.

Head localisation within the MEG helmet was achieved by measuring the locations of three energised electromagnetic coils, taped to the head, using a magnetic dipole fit. The positions of the coils relative to the subject's head shape were measured prior to acquisition using a 3D digitiser (Polhemus Isotrack). A structural MR image was acquired for each subject using an MPRAGE sequence on a Philips Achieva 3 T (1 mm^3^ resolution, *TR* = 8.3 ms, *TE* = 3.9 ms, *TI* = 960 ms, a shot interval of 3 s, *FA* = 8^∘^, and a SENSE factor of 2). Each structural scan was registered to the MNI152 standard brain template. The location of the MEG sensors relative to each subject's brain anatomy was found by matching the digitised head-shape to a scalp surface extracted from the MNI template and transformed (with 12 degrees of freedom) into the space of the subject's structural scan. Two subjects' data were discarded at this stage because of poor alignment. All further source-space analysis was carried out in MNI space.

Sensor data were down-sampled to 200 Hz. Any channels or segments of data showing obvious artefacts were removed following visual inspection. ICA Hyvärinen (1999) was used to further remove artefacts from the data as follows: sensor data were decomposed into 150 temporally independent components; artefact components were manually classified as relating to eye blinks (a high kurtosis, with a value over 20, and a repetitive blink structure over time), cardiac sources (strong resemblance to typical electrocardiogram signals and a kurtosis over 20) or mains interference (dominant 50 Hz component in the frequency spectrum); the artefact components were then subtracted out of the sensor data Mantini etal. (2011), which were subsequently band-pass filtered to 4–30 Hz.

### Specification of regions of interest

Separate cortical parcellations were employed for the analysis of the simulated and real data, shown in Figs. 2B and 5A. ROIs were defined for the simulated data by selecting a subset of 19 of the ROIs in the Harvard–Oxford cortical brain atlas, available in FSL, and splitting each into two lateral halves to create 38 binary ROIs. For the analysis of the resting-state data, a parcellation based on functional data was desired, which would define regions of interest based on functional specificity. We sought a parcellation which would allow us to investigate connections between and within both hemispheres and between individual, contiguous regions. We took a 100-dimensional group-spatial-ICA decomposition from resting-state fMRI scans of the first two hundred subjects in the Human Connectome Project database Van Essen etal. (2013), providing a set of spatial maps with highly local functional areas although mostly with bilateral distributions. We lateralised each component, and ran the resulting maps through a cluster-identifying algorithm, thresholding at *z* = 3.1, and retaining the cluster from each map with the maximum peak intensity. We then selected 38 of these clusters which provided a largely non-overlapping spanning of the cortex, choosing where possible pairs of ROIs, matched in each hemisphere. The z-statistic weightings of the spatial maps which survived the cluster thresholding were retained within the ROIs.

### Data processing

The same pipeline was used to process both simulated and real datasets.

Dipole sources on an 8 mm uniform grid were reconstructed using a scalar beamformer Van Veen etal. (1997), Robinson and Vrba (1999), Woolrich etal. (2011), a spatial filtering technique which uses a minimum-variance constraint to reconstruct signals at the locations of interest, while suppressing power from other locations. The data covariance matrix, used to estimate the beamformer reconstruction weights, was estimated from the entirety of each subject's data, and regularised by reducing the data dimensionality with principal components analysis, retaining between 260 and 264 components.

We represent the behaviour of each ROI with a single time-course, obtained using principal components analysis. The reconstructed sources within each ROI were first bandpass-filtered: the simulated data were analysed over a 4–30 Hz band, and the real MEG data within the alpha-band (8–13 Hz) and beta-band (13–30 Hz), chosen because many RSNs present well in these frequency ranges Brookes etal. (2011). Each ROI was normalised to have a positive peak hight of unity. The coefficients of the principal component accounting for the majority of the variance of the voxels within the ROI, weighted by the ROI map, were then taken as an appropriate representation of source activity for that region.

Signal leakage with zero temporal lag, induced by the beamforming process, was removed by symmetrically orthogonalising all ROI time-courses (Section 2.1). Following previous analyses Brookes etal. (2012b), Luckhoo etal. (2012), the power envelopes of these corrected ROI time-courses were then found by taking the absolute value of their Hilbert transform, low-pass filtering to 0.5 Hz and downsampling to 1 Hz.

### Connectivity analysis

Linear correlation and partial correlation (Eqs. (9)and (10)) analyses were performed on the down-sampled power envelopes of the corrected ROI time-courses of each subject or simulation. We chose the strength of regularisation applied to the partial correlation matrix for each subject using 10-foldwithin-subjectcross-validation, where the optimal λ was that which minimised the corrected Akaike information criterion Burnham and Anderson (2002), an information-theoretic model selection criterion which maximises a model's goodness of fit while penalising complexity. After the initial parameter selection, the search grid for λ was finessed three times.

Correlation values were converted to Z-scores using Fisher's transformation. In order to test the null hypotheses that there were no correlations between pairs of variables, the Z-scores for each edge were scaled such that the distributions of edge scores would be standard normal under fulfillment of the null. We computed the scaling as the standard deviation of an empirical null correlation distribution, generated by simulating several iterations of a null, uncorrelated Gaussian dataset, sharing the same number of nodes and temporal smoothness (modelled using the first auto-regressive coefficient) as the ROI time-courses, but with no spatial leakage, and taking correlations and unregularised partial correlations between the down-sampled, band-limited power envelopes of these data.

Group inference on the network structure was performed using a fixed-effects analysis testing the significance of the mean, over all subjects, of the standardised full or partial correlation Z-scores between each pair of ROIs. (We use fixed effects because we are not primarily interested here in subject variability, which would likely be poorly estimated from only eight subjects. The fixed effects analysis is telling us about the strength of the group-average effect.) False positives were controlled for the group at the network level using a false decision rate threshold.

### Method comparisons

The source-leakage correction method outlined above was compared to two alternatives. Firstly, not employing any correction at all. Secondly, a pair-wise orthogonalisation procedure, which employs the pair-wise orthogonalisation steps previously used for voxel-wise analyses Brookes etal. (2012b), Luckhoo etal. (2012) on the ROI time-courses. This works as follows: one ROItime-course is regressed from the other, power envelopes are found for the corrected time-courses, and their correlation computed. To estimate partial correlations between the same two nodes, the corrected time-course for each is regressed from all other ROI time-courses; power envelopes are found over all ROIs and the partial correlation between the power envelopes of the two ROIs of interest is computed. This procedure is iterated over all pairs of ROIs. Different correlations are found between each pair depending on which is chosen as the seed (i.e.,which ROI time-course is regressed from the other). The two correlations are averaged to produce the final estimate. Developing an approach to regularise these partial correlation estimations, using penalised regression approaches, is not trivial and is not relevant here; we restrict our comparison between the multivariate source-leakage correction and this pair-wise orthogonalisation procedure to unregularised estimates of partial correlations.

This comparative pair-wise procedure is significantly more computationally costly: to perform the symmetric orthogonalisation and cross-validated computation of regularised partial correlations between 38 node time-courses of one of our resting-state datasets took 70 s on a Macbook Pro with a 2.8 GHz Intel Core i7 processor and 16 GB of RAM; performing the orthogonalisation and calculation of (unregularised) partial correlations in a pair-wise fashion took 2000 s.

## Results

### Simulated data

The performance of our symmetric source-leakage correction method for ROI network connectivity analyses (Section 2.1) was assessed using a set of fifteen simulated experiments, with five networked dipoles, at an SNR of 1.0. The network structure and dipole locations are shown in Fig. 2 and the simulations are described in the Methods.

**Fig. 3:**
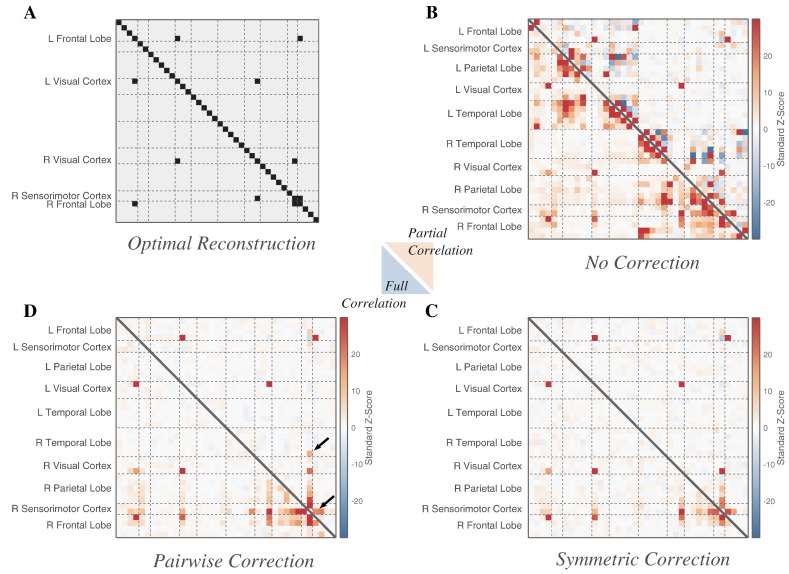
Reconstruction of a five-dipole network: Comparison of ability to infer a simulated network structure under different corrections for source leakage. Five connected dipoles were simulated in the left and right occipital lobes, the right and left frontal lobes, and the right somatosensory cortex (see Fig. 2), for 15 experiments of duration 10 min, at an SNR of 1.0. Group-level z-statistics for the full and partial correlations between the power time-courses of 38 cortical ROIs (Fig. 2B) are shown, after the application of corrections for source leakage. We combine two metrics into one heat map with full correlations below the diagonal and (unregularised) partial correlations above. We compare uncorrected data (B), data adjusted using the symmetric multivariate correction presented in this paper (C), and data corrected for zero-lag correlations pairwise between ROIs (D). C is the only set of matrices which can be thresholded to achieve perfect reconstruction. The higher arrow highlights an example false positive, and the lower a weak true positive with a neighbouring strong false positive (see text). A symmetrical version of the simulated network matrix is shown (A), indicating the relationships between ROIs which would constitute a perfect network discovery for these (undirected) network analysis methods.

Fig. 3 compares group-level estimates of the networked behaviour of the 38 ROIs in the brain, showing both the correlations and partial correlations between the power envelopes of all ROIs. We compare our proposed symmetric multivariate leakage correction to other forms of analysis: to not using a correction at all, and a pair-wise orthogonalisation procedure of ROI time-courses, an extension of a previously-employedvoxel-wise correction for seed-based correlation analyses (see Section 3). We did not attempt to regularise the partial correlations computed for this pair-wise method, which would require a significant increase in algorithmic and computational complexity; in this figure we display only unconstrained partial correlations to compare like with like (λ = 0).

Using both correction methods, we find much stronger suppression of local source spread, and better identification of the true network structure (as assessed with both full and partial correlation), compared with the results when no correction was applied. The partial correlation matrices, being estimates of the direct connections in the network, provide in general much better discrimination of the underlying network than the correlation matrices. (Neither method attempts to assign a direction to the functional connections.) While the spurious correlations present between the envelopes of the uncorrected ROI time-courses are nearly eliminated by the orthogonalisation methods, the correlations (marginal and partial) between ROIs corresponding to “true” edges are also to some extent weakened.

If we compare the symmetric multivariate correction (Fig. 3C) against the pair-wise orthogonalisation (Fig. 3D), we find the latter performs less well in suppressing artificial correlations, particularly among the full correlations. The partial correlations between these pair-wise corrected ROIs identified one additional false-positive network edge (indicated by the upper arrow), and a reduced correlation on a true network edge (lower arrow; with adjacent, stronger, false-positive) when compared to the symmetric correction, given an overall-optimal thresholding.

**Fig. 4:**
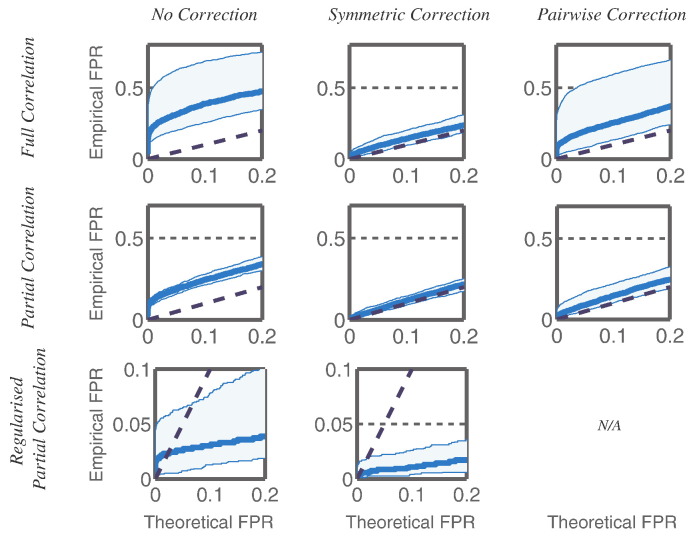
Control of false positives during network reconstruction. In each of fifty datasets, thirty-eight dipoles were simulated, of which five had activities driven by a directed network (Fig. 2D) and the rest were uncorrelated, and placed at random within 38 cortical ROIs (Fig. 2B), one dipole within each ROI. For each experiment, the full and partial correlations (calculated with and without regularisation of the precision matrix) between the band-limited power envelopes of the ROI time-courses were computed and used to infer the network structure between ROIs. For each of these metrics, we compare the expected false positive rate (FPR) for single edge detections against the empirical FPR as a measure of how well the spurious correlations between ROIs, introduced during source reconstruction, are removed by three orthogonalisation methods under test: applying no correction for spatial leakage (left column), applying the symmetric multivariate correction presented in this paper (middle column) and applying a pairwise orthogonalisation approach to the ROI time-courses (right column). Each graph plots the empirical FPR against the expected FPR as the threshold for defining network edges is moved. The solid blue line indicates the median behaviour over the fifty runs; the shaded background covers the central 95% of the data. An algorithm to combine the pairwise correction with regularised partial correlation has not been developed.

To understand if this behaviour was reproducible over a range of different dipole locations we extended our simulation. We generated fifty new datasets, with a reduced SNR of 0.4, in which each of the five dipoles was placed randomly in one of the 38 ROIs used for the analysis. We also placed a dipole in each of the remaining ROIs, and stimulated these individually with identically-generated but uncorrelated inputs to those driving the five networked dipoles. We then followed the same analysis procedure to arrive at correlation and partial correlation matrices for each of the fifty experiments, between all 38 ROIs, after applying each of the three source leakage correction methods. We investigated the extent to which each method removed spurious correlations between ROIs by comparing the false positive rates (FPR) for single-edge detections with those expected under a perfect correction for source leakage over a range of thresholds (Fig. 4).

We find that inference on network edges after applying the symmetric multivariate correction (Fig. 4, middle column) closely follows the expected false positive rates, with full correlation performing worse than partial correlation (a one-tailed Wilcoxon signed rank test for a difference in the median observed FPR, conducted at an expected FPR of 0.05 to match conventional thresholds, was significant, *p* < 10^− 3^), and dramatically improves upon these rates when regularisation is applied to the partial correlation matrix. Applying no leakage correction (Fig. 4, left column) leads to a much higher proportion of false positives, although regularisation of the partial correlation matrix is able to suppress many of the edges, reducing the false positive rate dramatically, although not to the same extent as for the corrected data. (Optimised regularisation strengths were similar: λ = 3.1 ± 1.1 × 10^− 2^ and λ = 1.1 ± 0.6 × 10^− 2^, for the uncorrected and corrected data respectively.) Correcting for source leakage in a pairwise manner (Fig. 4, right column), is less effective at removing false positives than the full multivariate correction, although when the network estimation is based on the partial rather than full correlations, this discrepancy is much reduced (yet still significant: a Wilcoxon signed rank test for the median of the observed pair-wise corrected FPR values being greater than the symmetrically corrected FPR values, conducted at an expected FPR of 0.05, was significant, *p* < 10^− 3^).

### Resting-state data

**Fig. 5:**
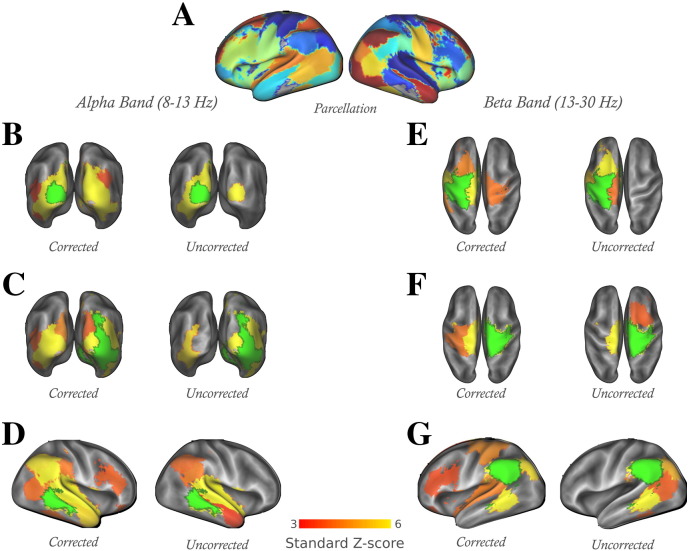
Group-level resting-state direct functional connections derived from eight subjects, inferred with and without the application of a multivariate spatial leakage correction between ROIs. Surface plots in the alpha-band (8–13 Hz, B–D) and beta-band (13–30 Hz, E–G) show direct functional connections between a single seed ROI (green) and the remaining 37 ROIs derived from an fMRI ICA decomposition. The chosen seed ROIs are in the visual (B and C)and sensori-motor (E and F)cortices, the left parietal (G)and right temporal (D)lobes; these plots correspond to the appropriately indicated columns in the network matrices of Supplementary Fig. 2. The colour scale indicates inference at group-level of individual regularised partial correlation z-statistics. All the surface plots have been thresholded at a 5% false discovery rate (*z* = 3.4 for the alpha band and *z* = 3.6 for the beta-band, in both corrected and uncorrected datasets). A surface rendering of the 38 ROIs is given for reference (A). Group-level resting-state direct functional connections derived from eight subjects, inferred with and without the application of a multivariate spatial leakage correction between ROIs. Surface plots in the alpha-band (8–13 Hz, B–D) and beta-band (13–30 Hz, E–G) show direct functional connections between a single seed ROI (green) and the remaining 37 ROIs derived from an fMRI ICA decomposition. The chosen seed ROIs are in the visual (B and C) and sensori-motor (E and F) cortices, the left parietal (G) and right temporal (D) lobes; these plots correspond to the appropriately indicated columns in the network matrices of Supplementary Fig.2. The colour scale indicates inference at group-level of individual regularised partial correlation z-statistics. All the surface plots have been thresholded at a 5% false discovery rate (*z* = 3.4 for the alpha band and *z* = 3.6 for the beta-band, in both corrected and uncorrected datasets). A surface rendering of the 38 ROIs is given for reference (A).

Functional connectivity was analysed for alpha- and beta-band oscillations between thirty-eight fMRI-derived ROIs (Fig. 5A) in resting-state MEG recordings from eight subjects, both with and without the application of our proposed symmetric multivariate source leakage correction. The connectivity between ROIs was modelled using an undirected Markov network with Gaussian variables (Eqs. (9)and (10)), applied to the down-sampled power envelopes of the ROI time-courses in the alpha band (8–13 Hz) and beta band (13–30 Hz). We estimated the presence and strength of edges in the network using a group-level analysis of the regularised subject-specific partial correlation matrices between the ROIs. (Optimised regularisation strengths were of similar magnitude forthe uncorrected and corrected data: λ = 3.2 ± 1.3 × 10^− 2^ and λ = 2.0 ± 1.1 × 10^− 2^ respectively for the alpha band; and λ = 2.2 ± 0.7 × 10^− 2^ and λ = 1.6 ± 0.9 × 10^− 2^ for the beta band.)

Our estimated networks for functional activity within these two bands, both with and without the correction for source leakage between ROIs, are shown in Fig. 5 and Supplementary Fig. 2. The surface plots show direct connections to and from individual ROIs, with a 5% false discovery rate (FDR) threshold applied (*z* = 3.4 for the alpha band and *z* = 3.6 for the beta-band, in both corrected and uncorrected datasets). These reveal strong clusters of short-range connections which clearly relate to regions well known as participating in independent networks: in the beta-band, the sensorimotor and parietal regions; in the alpha band, the visual network and local temporal lobe activity.

With the application of the symmetric source leakage correction between ROIs, the group-level networks, thresholded at a 5% FDR, showed an increased number of bilateral connections within the alpha-band visual network (although the net increase in un-thresholded cross-hemisphere edge-strengths within the 6 ROIs spanning the visual cortex was insignificant, median difference Δ*z* = 1.4, 95% CI [− 0.4, 2.9], *p* = 0.055 [for this and all estimates of visual alpha-band and sensorimotor beta-band connectivity differences in this section, the median difference with 95% confidence intervals is given by the Hodges–Lehmann estimator, associated with the Wilcoxon signed-rank test, together with a p-value on the same test with no adjustment for multiple comparisons] and there was no clear difference in overall edge-strength between these ROIs, Δ*z* = 1.2, 95% CI [− 4.8, 2.3]). The beta-band sensorimotor network emerged more cleanly as an independent cluster at the 5% FDR threshold, with stronger connections and reduced interference with more frontal regions (more generally, the median increase in un-thresholded edge strengths between 5 ROIs covering the beta-bandsensorimotor network was Δ*z* = 1.6, 95% CI [0.6, 2.5], *p* = 0.004, with the purely bilateral connections showing an insignificant change, Δ*z* = 1.3, 95% CI [− 0.3 3.1], *p* = 0.063). Direct connections between frontal and temporal lobes (alpha band, right hemisphere) and within the left fronto-parietal network (beta-band) also emerged at this threshold. By contrast, without the correction, direct connections between ROIs tend to be dominated by links to neighbouring regions, bilateral connections within the motor and visual networks were inhibited, and there were no edges linking ROIs in the frontal and parietal lobes. Some limited connectivity between neighbouring ROIs in the frontal lobe were present in the uncorrected data in both frequency bands; these were largely eliminated when the correction was applied.

**Fig. 6:**
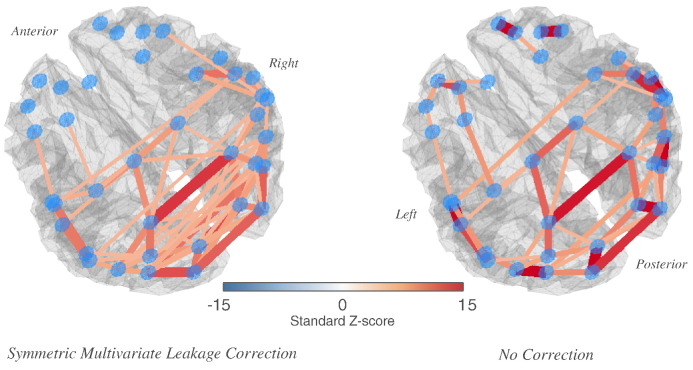
Alpha-band resting-state network structure surviving the 5% false discovery rate correction for multiple comparisons. A Comparison of network edges inferred for alpha-band (8–13 Hz) resting-state oscillations shows a much more densely connected visual network once the correction for source leakage is applied. Edges are shown as joining centres of mass of each ROI, with the colour scale and edge thicknesses indicating the group-level inference on regularised partial correlations between power envelopes of ROI time-courses. Only edges above the 5% false discovery rate thresholds are shown (*z* = 3.4 for both the corrected and uncorrected networks). (We recommend consulting Fig.1 in the supplementary information, a video version of this figure with a revolving camera. It provides a clearer view of the networks presented here.)

The full connectomes of the thresholded network matrices for the alpha band are shown in Fig. 6 (also as a video in Supplementary Fig. 1), with edges drawn between the centres of mass of each ROI. Again, for the symmetrically-corrected connectome as opposed to the uncorrected, there is a significantly higher prevalence of bi-lateral connections (median difference in edge strength for a bilateral connection within all 38 ROIs Δ*z* = 0.11, 95% CI [0.08, 0.16], *p* < 10^− 3^).

**Fig. 7:**
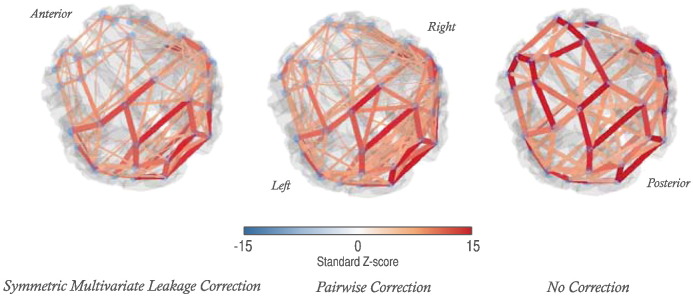
Alpha-band (8–13 Hz) resting-state partial correlations, with a comparison between orthogonalisation methods. Partial correlation, inferred without any regularisation to sparsity, yields denser connectomes (c.f. Fig. 6) and may be noisier than regularised estimates. In this analysis, there is little to differentiate the two leakage correction methods. Edges are shown as joining centres of mass of each ROI, with the colour scale and edge thicknesses indicating the group-level inference on regularised partial correlations between power envelopes of ROI time-courses. Only edges above the 5% false discovery rate thresholds are shown (*z* = 3.2, 3.4 and 3.3 respectively for the symmetrically-corrected, pairwise-corrected and uncorrected networks).

Lastly, in Fig. 7 we present, for comparison, the partial correlations between all 38 ROIs, thresholded at a 5% FDR. Estimating direct network connections using partial correlations without regularisation is noisy; here we merely observe the similarity between partial correlations estimated after the pair-wise and symmetric orthogonalisation processes. Full correlation, partial and regularised partial correlation matrices in the alpha and beta-bands for this dataset are available in the supplementary information.

## Discussion

We have presented a method for removing the confounding effects of source leakage before performing a correlation-based network analysis between regions of interest in source-reconstructed MEG data. Our approach corrects the time-courses of each ROI using a symmetric, multivariate orthogonalisation step before inferring direct network edges between ROIs from the *L*_1_-regularised partial correlations between the band-limited power envelopes of the corrected time-courses. The orthogonalisation step is optimal in the sense that it minimally displaces the set of ROI time-courses from their uncorrected forms.

Our work extends previous leakage correction methods Brookes etal. (2012a), Hipp etal. (2012) which, with one exception Brookes etal. (2014), were only appropriate for enabling correlation analyses between pairs of voxels in turn. Any multivariate analysis can now be performed on corrected ROI time-courses—possibilities include multivariate auto-regressive models or hidden Markov models Baker etal. (2014).

Our approach shares a limitation of these foundation methods, that the correction for leakage may not be perfect when sources are not Gaussian. This has been addressed in more detail in the appendix of Brookes etal. (2014). More generally, this method is limited by the compartmentalisation of brain function into a set of ROIs with only a single time-course each. On the one hand, using too few ROIs to capture the complexity of brain function risks a misrepresentation of the true network dynamics. Here, we have presented results using 38 ROIs from an fMRI group ICA, which is perhaps a coarse rendering of cortical network structure; future studies could explore the effectiveness of this technique using higher-dimensional parcellations. On the other hand, dividing into several ROIs part of the cortex that largely functions as a single coherent functional unit (given the spatial resolution and point spread function of the source reconstruction method), will cause those ROIs to be described by very similar time-courses. This situation is problematic for any network inference which partials out nodes, including partial correlation, as the partialling of one node against another with a shared time-course prevents the robust discovery of connectivity between their joint cortical unit and the rest of the brain. Under a full correlation analysis, the multivariate leakage correction proposed here could still create similar signal cancellation properties if two nodes are dominated by the same signal with zero-lag. As a result, whether or not a partial network analysis is performed, using ROIs which are too small or overlapping should be avoided: for example, the application of this method to compute dense or voxelwise connectomes would be inappropriate. One approach for mitigating this behaviour is to select ROIs using data-driven decompositions such as ICA, which differentiate cortical areas based on functional specificity. In particular, ICA decompositions of the same MEG dataset may provide cortical parcellations which reflect the spatial profile and resolution of the particular MEG measuring systems and reconstruction algorithms in use.

We have discussed the use of regularised partial correlation matrices for inference on a simple undirected Gaussian network model between ROIs. In line with many previous authors Smith etal. (2011), Marrelec etal. (2006), Duff etal. (2013) we find that partial correlation, whether with or without any imposed sparsity, is more effective at illuminating the true network structure than full correlation, which makes no attempt to discriminate between direct and indirect connections. Compare for example the network structures inferred by correlation and partial correlation methods in Fig. 3, and the elevated false-positive rate in the entire top row of Fig. 4 relative to the middle row. Fitting a model which encourages network sparsity in the partial correlation matrix is known to reduce noise and improve the estimation of correlations Duff etal. (2013), Varoquaux etal. (2010), Varoquaux and Craddock (2013), and in our simulations significantly improves network detection (where we are simulating a very sparse network), with strong suppression of unconnected edges to produce very low false positive rates.

Applying the multivariate leakage correction is effective, in simulation, at removing artefactual correlations between ROIs induced during the source reconstruction. In simulations of network dipoles at random locations through the cortex, inference on network structure using the partial correlations between the power envelopes of corrected ROItime-courses showed, on average, the expected control of false positives, whereas inference using uncorrected ROI time-series had a significantly higher rate of false detections. Inspection of the network matrices (Fig. 3) suggests that these are driven by false connections between proximate ROIs.

When we performed our network analyses on a resting-state dataset, applying the symmetric leakage correction to ROIs yielded denser connections within the alpha-band visual and beta-band sensorimotor networks, including a higher incidence of cross-hemispheric connections within these areas and a reduction in connections between the motor cortex and neighbouring regions in the frontal lobe (see Fig. 5). By contrast the networks inferred from the uncorrected ROIs are more dominated by local connections. Both of these observations have been previously noted in investigations of pair-wise source leakage corrections at the voxel level Brookes etal. (2012b), Hipp etal. (2012), Maldjian etal. (2014). Applying the correction also elucidated the detection, at a 5% false discovery rate threshold, of longer-rangebeta-bandfronto-parietal and alpha-bandfronto-temporal connections, which were less dominant than local connections in the uncorrected data. We expect these direct connections between frontal and parietal regions as the lateral fronto-parietal networks are common and stable independent components in fMRI decompositions of both MEG and fMRI data. Direct connections linking all of the regions known to be involved in the default mode network, such as the medial prefrontal cortex and inferior parietal cortex, were not evident in this dataset, either before or after corrections for source leakage. (Some electrophysiological studies identify this network, but with varying degrees of completeness compared to fMRI results. See, for example, Brookes etal. (2011), Baker etal. (2014), de Pasquale etal. (2010), Hillebrand etal. (2012), Duncan etal. (2013), Maldjian etal. (2014) and Wens etal. (2014)). In line with Hillebrand etal. (2012), we find an increased number of connections in regions associated with higher source power (occipital, parietal and temporal cortex in the alpha band; sensorimotor cortex in the beta-band). This may reflect the fact that corrected time-courses for ROIs with a higher SNR (and therefore better source specificity) are less rotated from their original positions than those with lower SNR. Lastly, we find it interesting (and encouraging) that the removal of zero-lag correlations from our data has actually increased the number of inferred direct connections (visible in Fig. 6).

In our simulations, we also evaluated an extension of previous bi-variatevoxel-wise corrections for source leakage to a pair-by-pair orthogonalisation process between ROIs. While it offered a reduction in the number of false positive edge detections over applying no correction at all, the control of the false positive rate was significantly poorer than desired for a leakage correction method (see Fig. 4). (When applied to our resting-state dataset, a comparison of the correlations and *un*regularised partial correlation matrices showed little to differentiate the results of the symmetric and bivariate orthogonalisation processes.) We hypothesise that the poorer performance of the pair-by-pair orthogonalisation is simply reflecting the failure of a bivariate correction process to account for the entrainment of non-zero lag connectivity between a pair of regions into their respective local neighbourhoods. We outline the process here and provide a simple mathematical treatment in the supplementary information; a nice discussion can be found in Palva and Palva (2012). Consider two ROIs, A and B, which are functionally connected in the sense that their time-courses exhibit strong cross-correlations even at non-zerotime-lags. The neighbours of B (the most proximate ROIs) may contain significant proportions of the signals in B due to the source leakage effect; this manifests itself as a strong correlation between B and the neighbours of B at purely zero lag. We may be able to prevent the false discovery of a connection between B and the neighbours of B by orthogonalising one with the respect to the other, which removes all zero-lag correlations. However, leakage from B into its neighbours can set up cross-correlations at non-zero lag between A and the neighbours of B. This “inherited connection” will not be accounted for by a pair-wise orthogonalisation procedure, but will by a multivariate process which considers the behaviour of all ROIs. Indeed, the partial correlations computed under the “pairwise correction” showed better false-positive control.

We further note not only that this pairwise approach is much more computationally demanding than the simple symmetrical orthogonalisation step we are proposing, but also that a method for finding regularised estimates of partial correlations has not yet been developed, is algorithmically more challenging and would create additional computational overheads, and that it is not straightforward to immediately apply any desired multivariate analysis method to the corrected time-courses. We therefore recommend the more effective and more efficient, symmetric approach for removing correlations between ROIs induced by source leakage. The number of ROIs which can effectively be corrected is limited by the rank of the data: our method cannot be applied to voxel-level network analyses, for example. Asking questions about the connectivity of more ROIs than there are independent time-series in the sensor data has dubious merit; but if these analyses are pursued, orthogonalisations between pairs of ROI time-courses may be the only feasible approach for limiting the effects of source leakage on amplitude correlation analyses.

Using a decomposition of the cortex into a set of ROIs to investigate functional network properties is common and profitable in fMRI. We believe that our approach for correcting ROI time-courses for source leakage correlations will enable a larger range of multivariate ROI analyses to be performed in the MEG community.

The following are the supplementary data related to this article.

SI figure 1 Alpha-band resting-state network structure surviving the 5% false discovery rate correction for multiple comparisons. Network edges inferred for alpha-band (8–13Hz) resting-state oscillations show a much more densely connected visual network once the correction for source leakage is applied (left), compared to inference on uncorrected regions of interest (right). Edges are shown as joining centres of mass of each ROI, with the colour scale and edge thicknesses indicating the group-level inference on regularised partial correlations between power envelopes of ROI time-courses for z between 0 and 15. Only edges above the 5% false discovery rate thresholds are shown (z=3.4 for both the corrected and uncorrected networks).

SI figure 2 Group-level resting-state network matrices derived from eight subjects, inferred with and without the application of a multivariate spatial leakage correction be- tween ROIs. Alpha-band (8–13Hz, left column) and beta-band (13–30Hz, right column) func- tional connectivity network matrices are inferred from a group-level average, at each edge, of individual regularised partial correlation z-statistics, between 38 ROIs derived from an fMRI ICA-based parcellation. Network matrices are presented both with (B and D) and without (A and C) a symmetric multivariate correction for source leakage applied to the ROI time-courses. Arrows above the matrices indicate the columns (i.e., seed ROIs) which are rendered as surface maps in figure 5.

SI figure 3 Group-level resting-state correlation matrices derived from eight subjects, inferred with and without the application of a symmetric, multivariate spatial leakage cor- rection between ROIs, and a pair-by-pair leakage correction. Alpha-band (8–13Hz, left column) and beta-band (13–30Hz, right column) functional connectivity correlation matrices are inferred from a group-level average, at each edge, of individual correlation z-statistics, between 38 ROIs derived from an fMRI ICA-based parcellation. Correlation matrices are presented both with (B and E) and without (A and D) a symmetric multivariate correction for source leakage applied to the ROI time-courses. Also presented (C and F) are correlation matrices for ROIs which have been orthogonalised in a pair-by-pair fashion before the computation of correlations.

SI figure 4 Group-level resting-state partial correlation matrices derived from eight subjects, inferred with and without the application of a symmetric, multivariate spatial leakage correction between ROIs, and a pair-by-pair leakage correction. Alpha-band (8–13Hz, left column) and beta-band (13–30Hz, right column) functional connectivity partial correlation matrices are inferred from a group-level average, at each edge, of individual correlation z-statistics, between 38 ROIs derived from an fMRI ICA-based parcellation. Partial correlation matrices are presented both with (B and E) and without (A and D) a symmetric multivariate correction for source leakage applied to the ROI time-courses. Also presented (C and F) are correlation matrices for ROIs which have been orthogonalised in a pair-by-pair fashion before the computation of partial correlations.

SI figure 5 Control of false positives during network reconstruction of a dense network. In each of fifty datasets, thirty-eight dipoles were simulated, of which fifteen had activities driven by a directed network (A) and the rest were uncorrelated, and placed at random within 38 cortical ROIs (figure 2B), one dipole within each ROI. For each experiment, the full and partial correlations (calculated with and without regularisation of the precision matrix) between the band-limited power envelopes of the ROI time-courses were computed and used to infer the network structure between ROIs. For each of these metrics, we compare the expected false positive rate (FPR) for detection of each edge against the empirical FPR as a measure of how well the spurious correlations between ROIs, introduced during source reconstruction, are removed by three orthogonalisation methods under test: applying no correction for spatial leakage (left column), applying the symmetric multivariate correction presented in this paper (middle column) and applying a pairwise orthogonalisation approach to the ROI time-courses (right column). Each graph plots the empirical FPR against the expected FPR as the threshold for defining network edges is moved. The solid blue line indicates the median behaviour over the fifty runs; the shaded background covers the central 95% of the data. An algorithm to combine the pairwise correction with regularised partial correlation has not been developed.

SI figure 6 Illustration of the effect of the symmetric orthogonalisation process on a time- course. We illustrate the effect of the symmetric orthogonalisation process and the pair-by-pair orthogonalisation on the envelopes of two time-courses simulated from the functional network of 38 nodes, with 5 connected to each other and the remainder independent, used to generate the data for figure 4. In black, is the amplitude envelope of the activity time-course for one randomly-chosen unconnected node. In A, in dashed orange, is the envelope of the same node after symmetrically orthogonalising all 38 nodes. In B, in dashed orange, is the envelope of the same node after orthogonalising using the Gram-Schmidt process to one other, randomly chosen node. For this example, the symmetrically-corrected envelope is closer to the uncorrected envelope than is the Gram-Schimdt-corrected version (Pearson’s R2 is 0.86 for the former and 0.76 for the latter).

## Footnotes

1 The probability of waiting *t* ≥ 0 seconds for a transition from one state, with meanoccupation time *τ* seconds, to the other is given by the exponential distribution, $p\left(t|\tau \right)=\frac{1}{\tau }e^{-t/\tau }$.
