# Supplementary figures and images for: A symmetric multivariate leakage correction for MEG connectomes
Source: Neuroimage. 2015 Aug 15;117:439–48. doi: 10.1016/j.neuroimage.2015.03.071 (PMC4528074; doi:10.1016/j.neuroimage.2015.03.071)

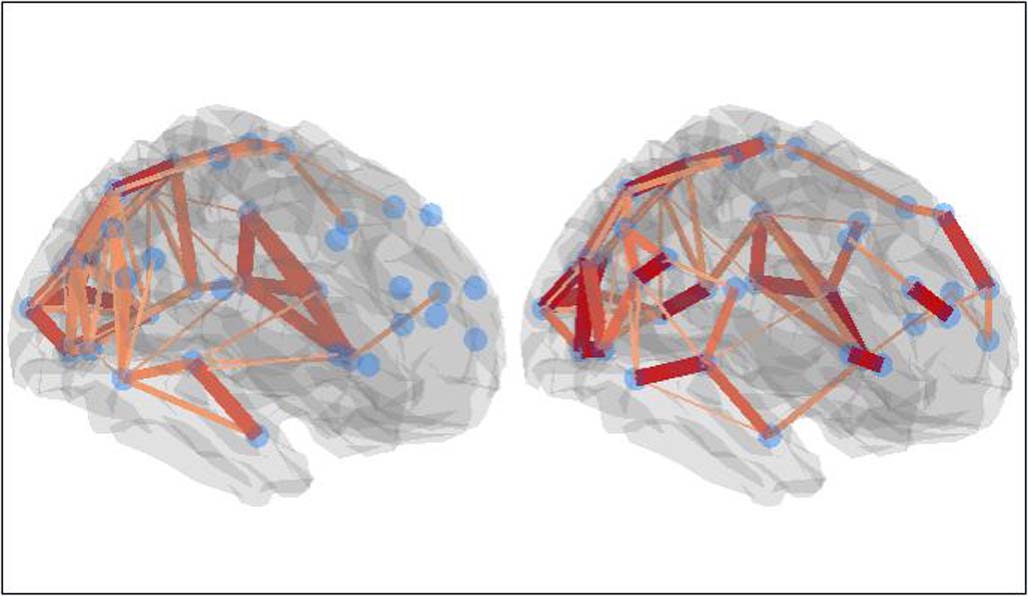

Supplement: SI figure 1 — Alpha-band resting-state network structure surviving the 5% false discovery rate correction for multiple comparisons. Network edges inferred for alpha-band (8–13Hz) resting-state oscillations show a much more densely connected visual network once the correction for source leakage is applied (left), compared to inference on uncorrected regions of interest (right). Edges are shown as joining centres of mass of each ROI, with the colour scale and edge thicknesses indicating the group-level inference on regularised partial correlations between power envelopes of ROI time-courses for z between 0 and 15. Only edges above the 5% false discovery rate thresholds are shown (z=3.4 for both the corrected and uncorrected networks). [file mmc1.jpg]

No Correction

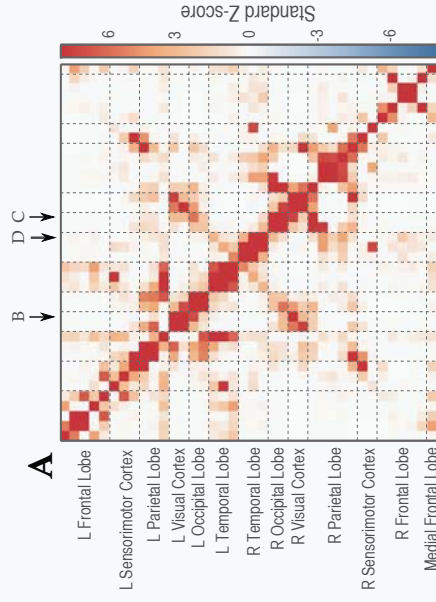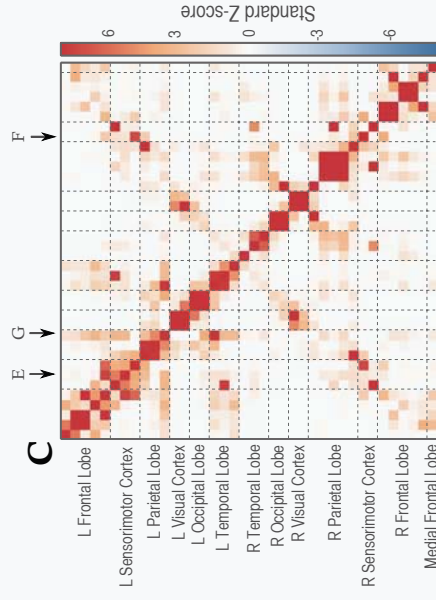

Symmetric Multivariate Leakage Correction

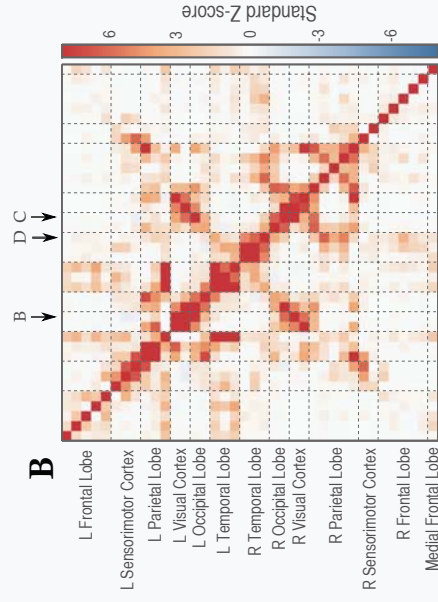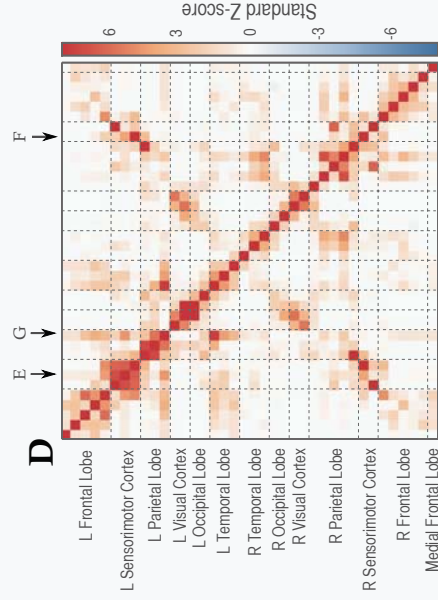

Alpha Band (8-13 Hz)

Beta Band (13-30 Hz)

Supplement: SI figure 2 — Group-level resting-state network matrices derived from eight subjects, inferred with and without the application of a multivariate spatial leakage correction be- tween ROIs. Alpha-band (8–13Hz, left column) and beta-band (13–30Hz, right column) func- tional connectivity network matrices are inferred from a group-level average, at each edge, of individual regularised partial correlation z-statistics, between 38 ROIs derived from an fMRI ICA-based parcellation. Network matrices are presented both with (B and D) and without (A and C) a symmetric multivariate correction for source leakage applied to the ROI time-courses. Arrows above the matrices indicate the columns (i.e., seed ROIs) which are rendered as surface maps in figure 5. [file mmc2.pdf]

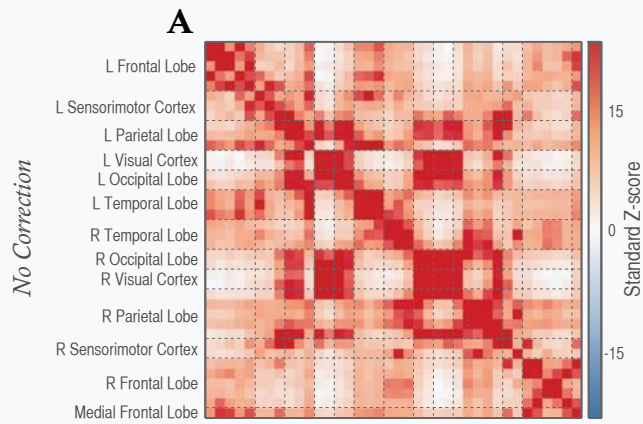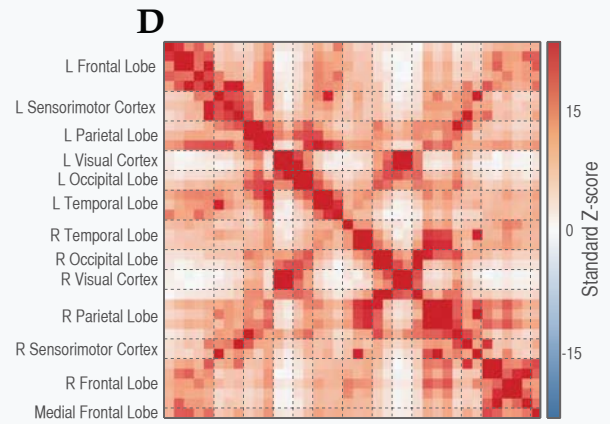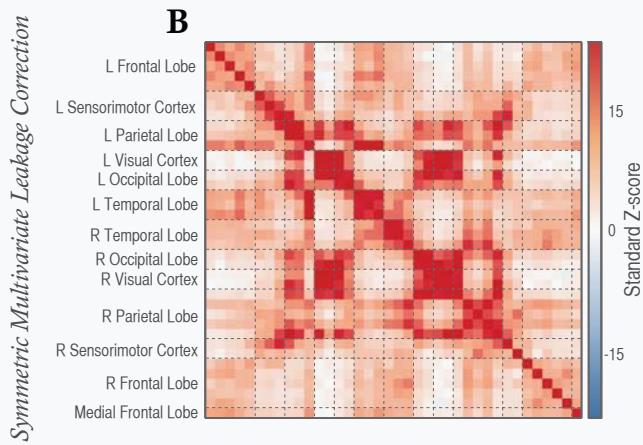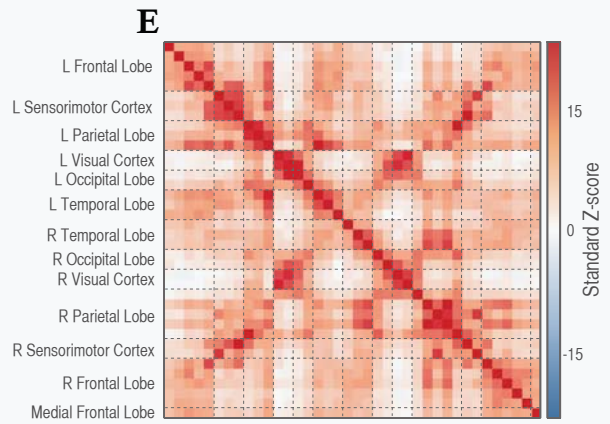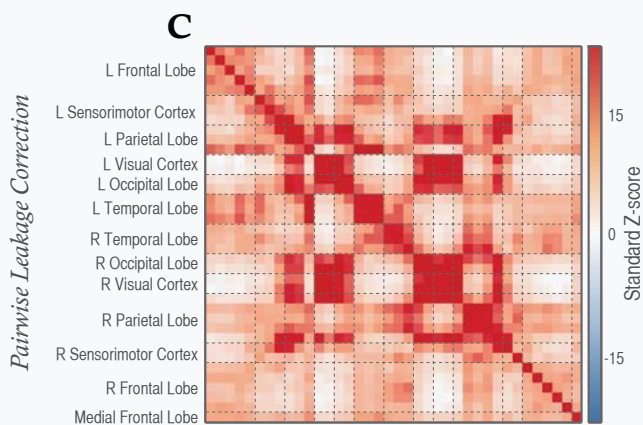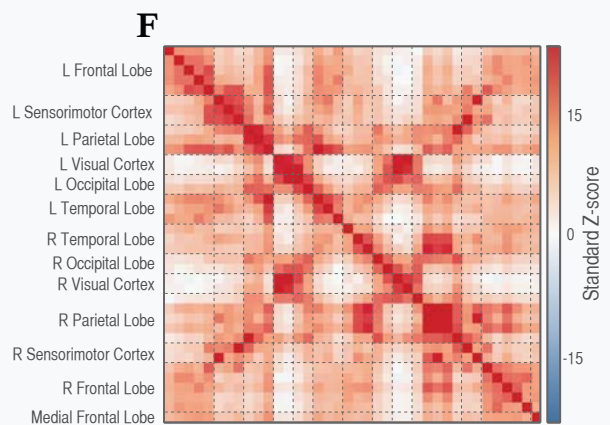

*Alpha Band (8-13 Hz)*

*Beta Band (13-30 Hz)*

Supplement: SI figure 3 — Group-level resting-state correlation matrices derived from eight subjects, inferred with and without the application of a symmetric, multivariate spatial leakage cor- rection between ROIs, and a pair-by-pair leakage correction. Alpha-band (8–13Hz, left column) and beta-band (13–30Hz, right column) functional connectivity correlation matrices are inferred from a group-level average, at each edge, of individual correlation z-statistics, between 38 ROIs derived from an fMRI ICA-based parcellation. Correlation matrices are presented both with (B and E) and without (A and D) a symmetric multivariate correction for source leakage applied to the ROI time-courses. Also presented (C and F) are correlation matrices for ROIs which have been orthogonalised in a pair-by-pair fashion before the computation of correlations. [file mmc3.pdf]

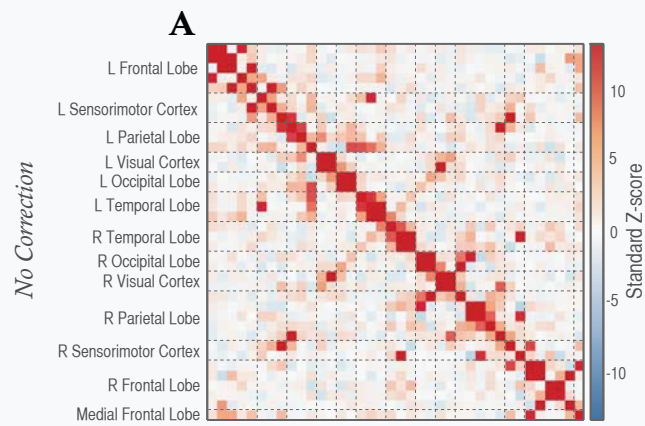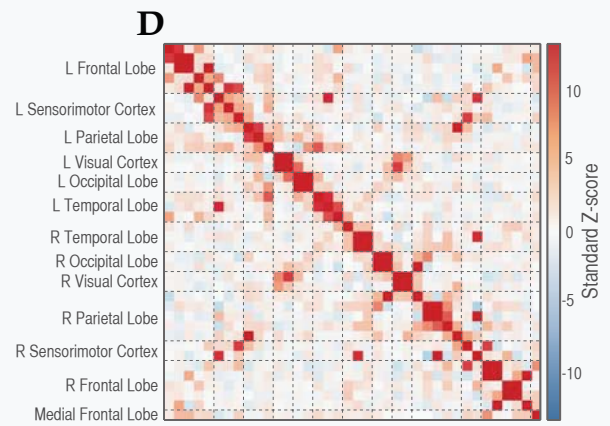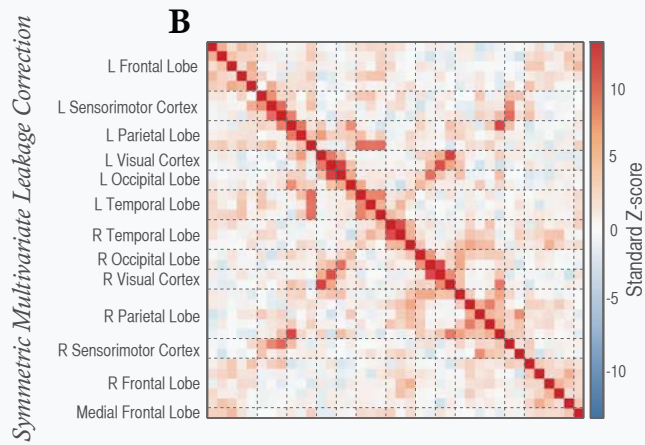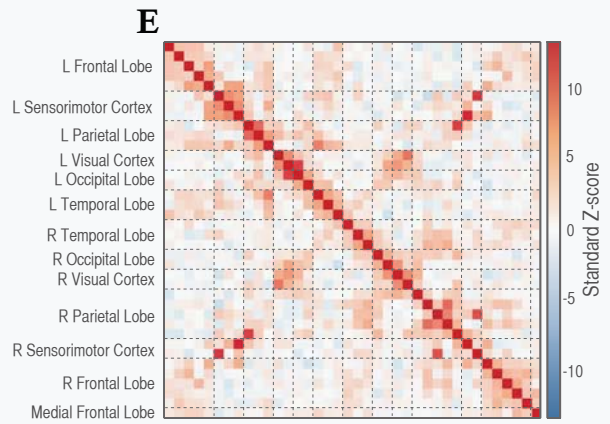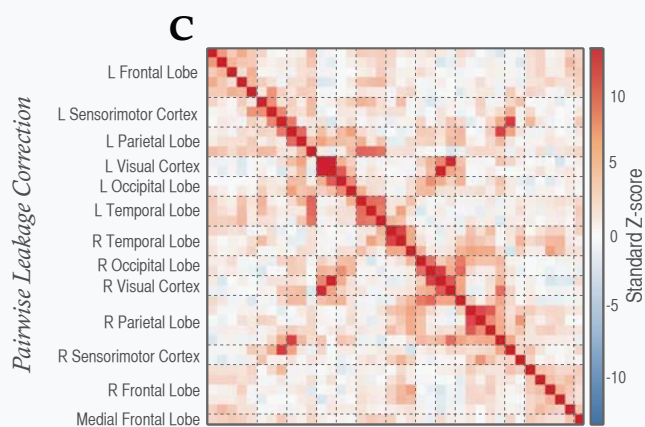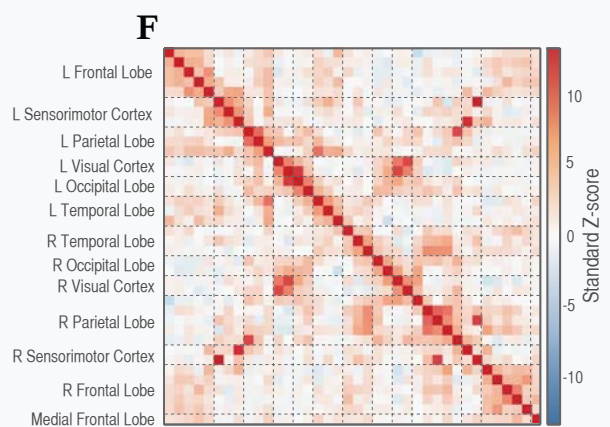

*Alpha Band (8-13 Hz)*

*Beta Band (13-30 Hz)*

Supplement: SI figure 4 — Group-level resting-state partial correlation matrices derived from eight subjects, inferred with and without the application of a symmetric, multivariate spatial leakage correction between ROIs, and a pair-by-pair leakage correction. Alpha-band (8–13Hz, left column) and beta-band (13–30Hz, right column) functional connectivity partial correlation matrices are inferred from a group-level average, at each edge, of individual correlation z-statistics, between 38 ROIs derived from an fMRI ICA-based parcellation. Partial correlation matrices are presented both with (B and E) and without (A and D) a symmetric multivariate correction for source leakage applied to the ROI time-courses. Also presented (C and F) are correlation matrices for ROIs which have been orthogonalised in a pair-by-pair fashion before the computation of partial correlations. [file mmc4.pdf]

A

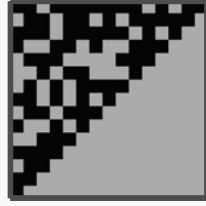

Network matrix for 15 connected,  
randomly placed, dipoles

B

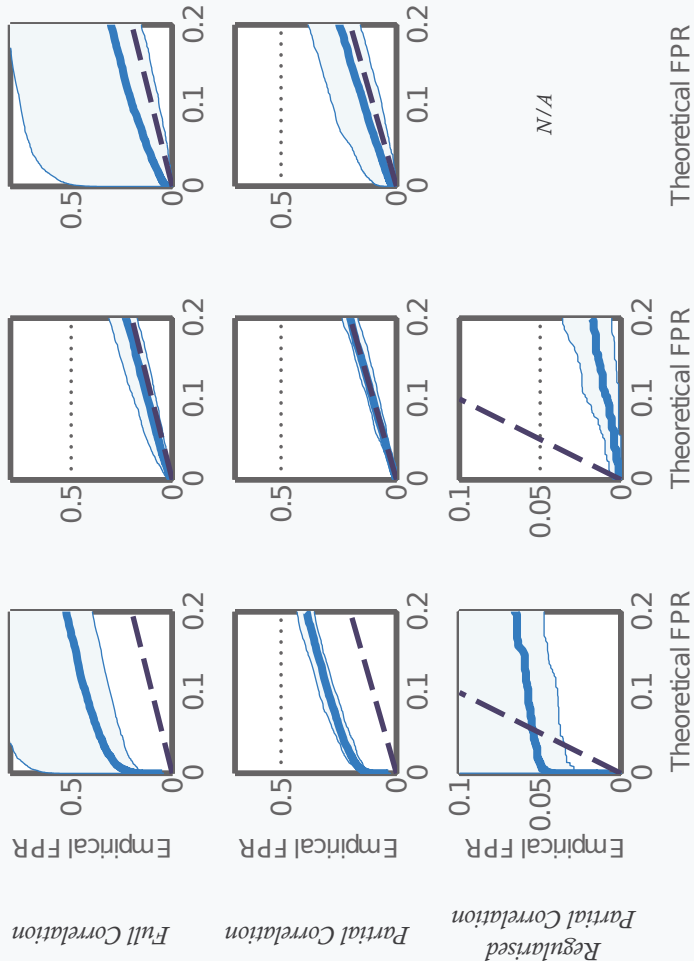

Supplement: SI figure 5 — Control of false positives during network reconstruction of a dense network. In each of fifty datasets, thirty-eight dipoles were simulated, of which fifteen had activities driven by a directed network (A) and the rest were uncorrelated, and placed at random within 38 cortical ROIs (figure 2B), one dipole within each ROI. For each experiment, the full and partial correlations (calculated with and without regularisation of the precision matrix) between the band-limited power envelopes of the ROI time-courses were computed and used to infer the network structure between ROIs. For each of these metrics, we compare the expected false positive rate (FPR) for detection of each edge against the empirical FPR as a measure of how well the spurious correlations between ROIs, introduced during source reconstruction, are removed by three orthogonalisation methods under test: applying no correction for spatial leakage (left column), applying the symmetric multivariate correction presented in this paper (middle column) and applying a pairwise orthogonalisation approach to the ROI time-courses (right column). Each graph plots the empirical FPR against the expected FPR as the threshold for defining network edges is moved. The solid blue line indicates the median behaviour over the fifty runs; the shaded background covers the central 95% of the data. An algorithm to combine the pairwise correction with regularised partial correlation has not been developed. [file mmc5.pdf]

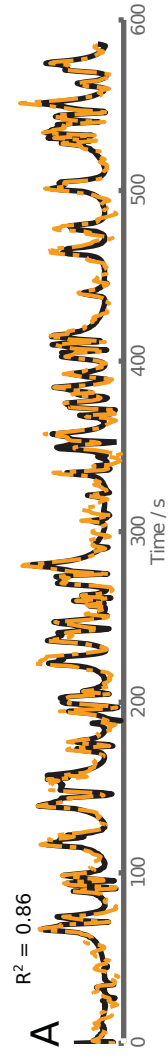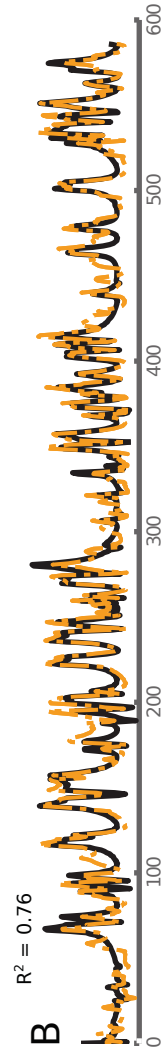

Supplement: SI figure 6 — Illustration of the effect of the symmetric orthogonalisation process on a time- course. We illustrate the effect of the symmetric orthogonalisation process and the pair-by-pair orthogonalisation on the envelopes of two time-courses simulated from the functional network of 38 nodes, with 5 connected to each other and the remainder independent, used to generate the data for figure 4. In black, is the amplitude envelope of the activity time-course for one randomly-chosen unconnected node. In A, in dashed orange, is the envelope of the same node after symmetrically orthogonalising all 38 nodes. In B, in dashed orange, is the envelope of the same node after orthogonalising using the Gram-Schmidt process to one other, randomly chosen node. For this example, the symmetrically-corrected envelope is closer to the uncorrected envelope than is the Gram-Schimdt-corrected version (Pearson’s R2 is 0.86 for the former and 0.76 for the latter). [file mmc6.pdf]
